# Phospholipase D regulates ferroptosis signal transduction in mouse spleen hypoxia response

**DOI:** 10.1590/1414-431X2023e13218

**Published:** 2024-03-04

**Authors:** Jiayang Wang, Ying Hu, Yuzhen Xu, Qifu Long, Cunlin Gu, Chaoqun Tang, Ru Wang, Sheng Yong

**Affiliations:** 1 Department of Basic Medicine, School of Medicine, Qinghai University, Xining, Qinghai Province, China

**Keywords:** Hypoxia, Spleen, Ferroptosis, Phospholipase D pathway, Transcriptomic, Proteomic

## Abstract

High-altitude hypoxia exposure can lead to phospholipase D-mediated lipid metabolism disorder in spleen tissues and induce ferroptosis. Nonetheless, the key genes underlying hypoxia-induced splenic phospholipase D and the ferroptosis pathway remain unclear. This study aimed to establish a hypoxia animal model. Combined transcriptomic and proteomic analyses showed that 95 predicted target genes (proteins) were significantly differentially expressed under hypoxic conditions. Key genes in phospholipase D and ferroptosis pathways under hypoxic exposure were identified by combining Gene Ontology (GO) and Kyoto Encyclopedia of Genes and Genomes (KEGG) enrichment analysis techniques. Gene set enrichment analysis (GSEA) showed that the differential gene sets of the phospholipase D and ferroptosis signaling pathways were upregulated in the high-altitude hypoxia group. The genes in the phospholipase D signalling pathway were verified, and the expression levels of *KIT* and *DGKG* were upregulated in spleen tissues under hypoxic exposure. Subsequently, the mRNA and protein expression levels of genes from the exogenous pathway such as *TFRC*, *SLC40A1*, *SLC7A11*, *TRP53*, and *FTH1* and those from the endogenous pathway such as *GPX4*, *HMOX1*, and *ALOX15* differentials in the ferroptosis signalling pathway were verified, and the results indicated significant differential expression. In summary, exposure to high-altitude hypoxia mediated phospholipid metabolism disturbance through the phospholipase D signalling pathway and further induced ferroptosis, leading to splenic injury.

## Introduction

The plateau environment has low pressure, low oxygen, cold dryness, and other adverse factors, and the body in this environment undergoes a series of physiological changes (1). Specifically, the highland hypoxic environment can directly affect iron metabolism (2). Under hypoxic conditions, the activity of intracellular proline hydroxylase decreases, and the hypoxia-inducible factor (HIF) protein cannot be hydroxylated, further blocking the degradation of the HIF's ubiquitin protease system (3), leading to a continuous increase in HIFs in cells and changes in iron metabolism (4). Simultaneously, hypoxia can stimulate the mitochondrial production of excessive reactive oxygen species (ROS) (5), promote the accumulation of lipid peroxidation of polyunsaturated fatty acids (PUFAs) in the cell membrane, and induce ferroptosis (6). Therefore, studying the mechanisms of ferroptosis and lipid metabolism under hypoxic stress is important.

The spleen is a blood storage organ and plays an important role in maintaining the number and function of blood cells (7). A high-altitude hypoxic environment increases the synthesis of circulating hemoglobin (8) and induces increased intracellular iron uptake in spleen tissues and abnormal iron deposition in spleen cords, leading to oxidative damage and triggering ferroptosis (9). Excess iron undergoes the Fenton reaction to produce ROS, disrupting the balance between oxidation and antioxidant activity. Consequently, lipid peroxidation occurs (10), which further affects immune function (11,12). However, how hypoxia induces ferroptosis and lipid metabolism disorders in the spleen, leading to spleen tissue damage in mice, requires further investigation.

Thus, this study aimed to establish a high-altitude hypoxia animal model at an altitude of 4200 m, conduct transcriptomic sequencing and tandem mass tag (TMT) quantitative proteomic association analysis on spleen tissues using multi-omics analysis technology, and integrate and screen the differentially expressed genes (DEGs) and differentially expressed proteins (DEPs). It also focused on changes in the 95 predicted target gene sets identified by the transcription-protein combination under exposure to high-altitude hypoxia. Subsequently, two pathways of ferroptosis and phospholipase D were screened, and the key genes and molecular mechanisms for ferroptosis and phospholipid metabolism disorder in spleen tissues under hypoxic exposure were clarified.

## Material and Methods

### Experimental animals and grouping

Specific pathogen-free healthy male C57BL/6 mice aged 6-8 weeks were purchased from the Experimental Animal Centre of the Medical Department of Xi'an Jiaotong University (experimental animal license numbers: SYXK (Shan) 2020-005, Xi'an). Mice were randomly divided into two groups (n=5/group). Mice in the control or plain spleen control (PSC) group were raised at the Experimental Animal Centre of the Medical Department of Xi'an Jiaotong University (400 m above sea level), and those in the experimental or high-altitude spleen test (HST) group were raised in an experimental animal room at Maduo County People's Hospital of Guoluo Tibetan Autonomous Prefecture, Qinghai Province (4,200 m above sea level). The temperature at the experimental animal room was 18-22°C with a humidity of 45-55%. After 30 days, mouse spleen tissues were aseptically collected and cryopreserved.

### Measurement of spleen index in mice

After fasting body weights were obtained, mouse spleen tissues were aseptically collected and weighed. The mouse spleen index was calculated as follows: spleen index = spleen mass (g) / body mass (g) × 100.

### Transcriptomic analysis

RNA-Seq quality assessment and sequence alignment primarily included the removal of reads with sequencing adapters from the original data or reads with lower sequencing quality and subsequent high-quality analysis of clean data. The index was constructed using HISAT2 software (https://daehwankimlab.github.io/hisat2), and the paired terminal clean reads were compared with the reference genome. The gene expression levels of the samples were analyzed using Pearson's correlation test.

### Proteomic analysis

Analysis was performed using a combination of a 1200-nm upgraded UHPLC LC system (EASY-nLC™; Thermo Fisher/LC140, Germany) and a mass spectrometer (Q Exactive™ HF-X; Thermo Fisher). According to the protein database, the result spectra of each run were searched separately using Proteome Discoverer software version 2.4 (Thermo Fisher). Furthermore, to improve the quality of analysis results, the retrieved results were also filtered using Proteome Discoverer software version 2.4. Peptide-spectrum matches with reliability >99% were considered credible peptide-spectrum matches, and credible peptide spectra and protein spectra were retained.

### Main reagents

The main reagents used in this study included TRIzol lysis buffer (Invitrogen, USA); reverse transcription kit: PrimeScript RT reagent kit with GDNA Eraser (RR 047A, Takara, Japan); qPCR kit: Tb Green Premix EX TAQ II (RR 820A, Takara); animal whole protein extraction kit (C510003, Sangon Biotech, China); BCA protein concentration assay kit (P0010, Beyotime Biotech, China); 5× loading buffer (P0015, Beyotime Biotech); SDS-PAGE gel preparation kit (P1200, Solar BioBeijing, China); SDS-PAGE electrophoresis solution (P00148, Beyotime Biotech); 10× EMT (D1060, Solar BioBeijing); methanol (CB/T693-1993, Shanghai Guangnuo Chemical Technology Co., Ltd., China); skimmed milk powder (D8340, Solar BioBeijing); 10×TBST buffer (powder) (T1087, Solar BioBeijing); nitrocellulose membrane (NC membrane) (37412133, PALL, USA); and ECL developer (34095, Thermo Fisher, USA).

### Combined transcriptomic and proteomic analyses

Based on the protein omics level, a *t*-test was used to analyze the quantitative results of the proteins, and those (P<0.05) for which the quantitative difference was significant were defined as DEPs. Transcriptome data relied on the FPKM (fragments per kilobase per million mapped fragments) quantitative value of genes, the corrected P-value was adjusted to control the error rate using Benjamini and Hochberg's methods, and |log2foldchange|>1 was used as the threshold for significant DEGs.

The Gene Ontology (GO, http://www.geneontology.org/) database consists of biological processes (BP), cellular components (CC), and molecular functions (MF). KEGG (Kyoto Encyclopedia of Genes and Genomes, http://www.kegg.jp/) is a database resource used at the molecular level to understand the high-level functions and utilities of cells, organisms, and ecosystems through high-throughput databases. Simultaneously, the annotated results were subjected to pathway-significant enrichment analysis and the P-value was calculated. P<0.05 indicated statistically significant enrichment.

### GSEA based on KEGG functional annotation

In this study, GSEA (http://www.broadinstitute.org/gsea/index.jsp) of the KEGG data set was performed using the GSEA tool provided by the Broad Institute, and the pathway enrichment results were screened with a P-value of <0.05 in an attempt to supplement the results of the previous functional enrichment analysis.

### RT-qPCR and western blotting experiments

RT-qPCR validation was performed using β-actin as an internal reference, and RNA from mouse spleen tissues was reversely transcribed into cDNA using the PrimeScript™ RT kit according to the manufacturer's instructions. The reaction volume was 10 μL for each reagent, 6.4 μL for RNase-Free H_2_O, 20 μL for cDNA, and 0.8 μL for primers before and after PCR. Three biological replicates were conducted, and three technical replicates were conducted for each biological replicate. The primer sequences used are listed in Supplementary Table S1.

Mouse spleen samples were removed and placed in a centrifuge tube equipped with a lysis buffer. The spleen was cut into pieces using a sterile surgical shear in a centrifuge tube and sonicated. The centrifuge was precooled to 4°C. The protein concentration was detected using the BCA method, and the protein loading concentration was quantified by adding the corresponding volume of enzyme-free water and loading buffer after calculation. The loading amount of 50 μg protein was unified, and after loading, electrophoresis (constant pressure 90 V) and protein transfer after electrophoresis (cross-flow 200 mA) were performed. After the NC membrane was washed once with TBST washing solution, the NC membrane was closed with 5% defatted milk for 2 h, during which time the primary anti-antibody was diluted in proportion and placed at 4°C for subsequent use. After blocking, the membranes were washed three times with TBST, incubated with the primary antibody (4°C overnight), and removed the next day. The secondary antibodies were incubated (room temperature, 90 min) after the membranes were washed with TBST washing solution (5 min/wash, six times + 10 min per time, once). After the membranes were washed again (6 min/wash, 5 times), they were incubated with ECL developer solution (protected from light) and imaged using the VILBER Fusion Solo Imaging Analysis System (France).

### Statistical analysis

Differences in the results were analyzed using SPSS version 18.0 (IBM Corp., USA), and two independent samples *t*-tests were used to test statistical significance. All results are reported as means±SD, and the difference was considered to be statistically significant at P<0.05.

## Results

### Detection of spleen index in mice

**Figure 1:**
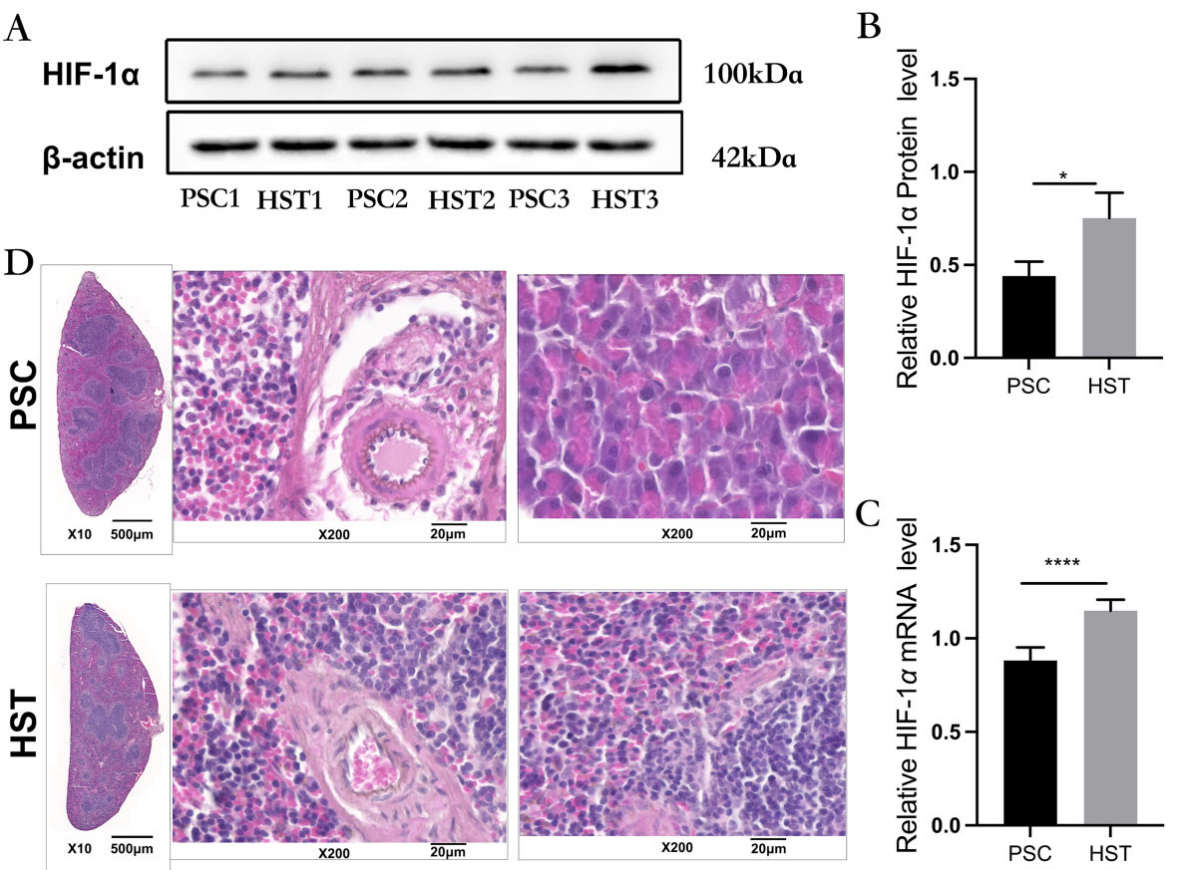
Expression of hypoxia-inducible factor (HIF)-1α in spleen tissues of mice in the high-altitude spleen test (HST) group and in the plain spleen control (PSC) group. **A**, Western blot analysis of HIF-1α protein expression. **B**, Quantitative analysis of HIF-1α protein expression with the β-actin as standard. **C**, mRNA expression analysis of HIF-1α. Data are reported as means±SD (n=3). *P<0.05, ****P<0.0001 compared with PSC (Student's *t*-test). **D**, HE staining of mouse spleen tissue (scale bar 20 μm).

After 30 days of exposure to high-altitude hypoxia, the mRNA and protein expression levels of *HIF-1α* were detected, and the expression of *HIF-1α* in spleen tissues was higher in the HST group than in the PSC group (Figure 1A-C), suggesting that the development of the highland hypoxia mouse model was successful. Compared with the PSC group, body weights and spleen index of mice in the HST group were significantly decreased (P<0.0001 and P<0.01) (Table 1). These results indicated that the weight of mouse spleen tissues were reduced under high-altitude hypoxia. HE staining revealed a blurred boundary between the splenic cortex and the medulla, a disorganized medulla, a change in the medullary ratio, and a large number of lymphocyte infiltrates could be observed in the HST group (Figure 1D). The results suggested that hypoxia exposure induced tissue damage in the mouse spleen.

**Table 1:** Detection of spleen index in mice.

Group	Body mass (g)	Spleen mass (g)	Spleen index (%)
PSC	22.710±0.552	0.0628±0.005	0.276±0.020
HST	19.030±0.270	0.045±0.003	0.235±0.012
*T*	13.370****	6.261***	3.912**

HST: high-altitude spleen test group; PSC: plain spleen control group. **P<0.01, ***P<0.001, ****P<0.0001 for difference between groups.

### Transcriptomic sequencing, data processing, and quality assessment

**Figure 2:**
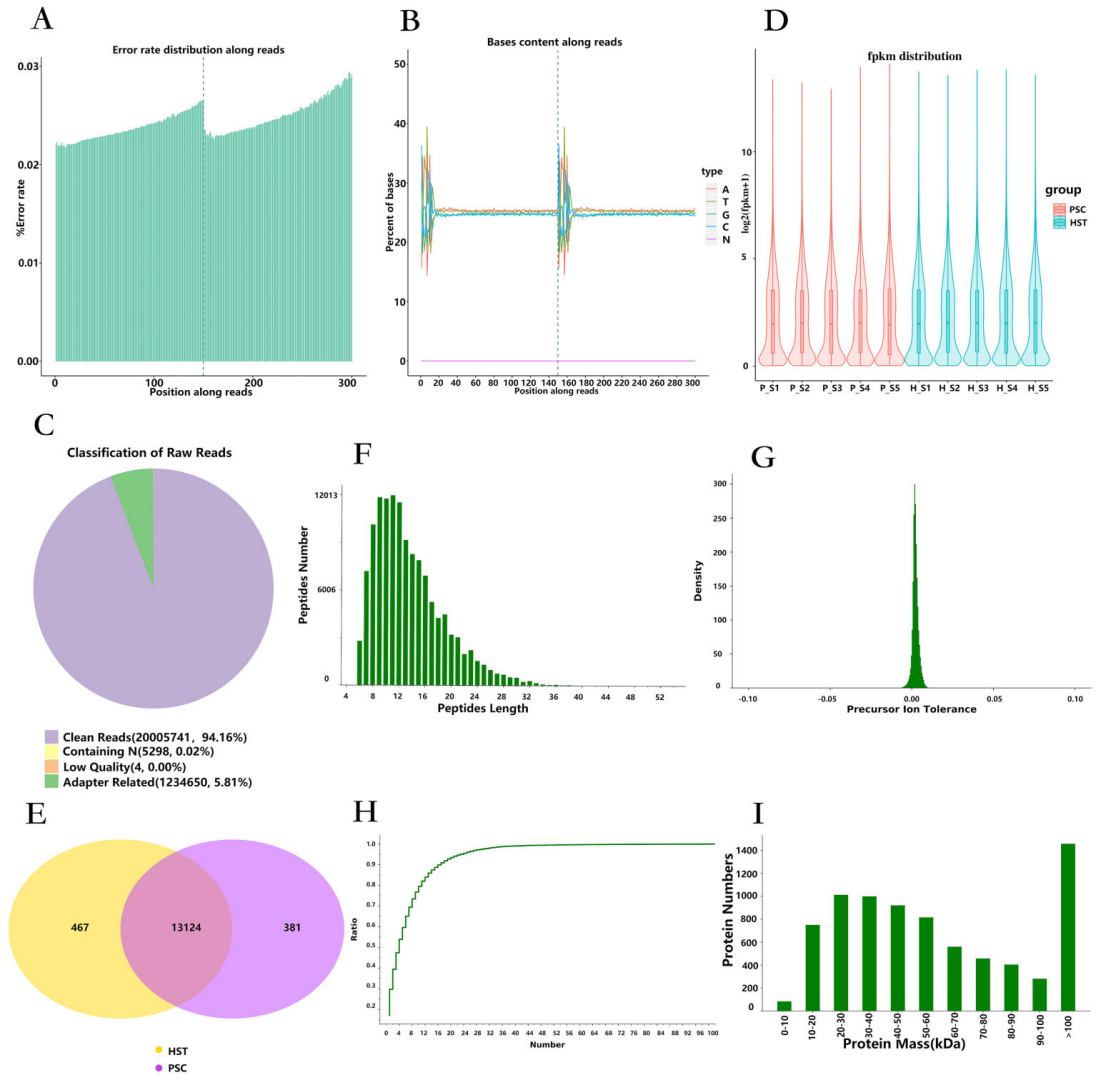
Transcriptomic sequencing of samples from the high-altitude spleen test (HST) group and in the plain spleen control (PSC) group. **A**, Sequencing data error rate distribution. **B**, GC content distribution. **C**, Sample sequencing data filtering. **D**, Sample gene expression distribution. **E**, Differential gene Venn diagram. **F**, Peptide length range distribution map. **G**, Parent ion mass tolerance distribution. **H**, Distribution map of the number of unique peptides in identified proteins. **I**, Protein molecular weight distribution. GC content: ratio of quinine and cytosine among the four bases of DNA.

Five biological repeat samples from the PSC and HST groups were sequenced using the Illumina sequencing platform (https://www.illumina.com/systems/sequencing-platforms.html) to construct a transcriptome library, and the quality of raw data was controlled (Table S2). The sequencing error rate of a single base position in RNA-Seq sequencing was less than 1% (Figure 2A). The 6-bp random primer used for the first few bases during reverse transcription normally fluctuated and then stabilized to a horizontal line (Figure 2B). A small number of reads with sequencing linkers or low sequencing quality were removed from the sequencing raw data to obtain clean reads, accounting for 94.16% (Figure 2C). After further calculating the FPKM of all genes, the distribution of gene expression levels in the PSC and HST groups was obtained, with an average log2(fpkm+1) of approximately 2 (Figure 2D). Subsequently, the overlapping of differential genes between the PSC and HST groups was compared with |log2FC|≥0 and P-adjustment <0.05. Overall, 13,505 and 13,591 differential genes were identified in the PSC and HST groups, respectively; of these, 13,124 differential genes were found in the two groups (Figure 2E). The above experimental results suggested that the results of transcriptome sequencing experiment were reliable, and there were more DEGs in mouse spleen tissues under high-altitude hypoxic stress in the two groups.

### Proteomic mass spectrometry data processing and quality control

Quality evaluation was performed based on the sequencing results of the protein groups. After the samples of the PSC and HST groups were detected by mass spectrometry, the identified peptide and protein information was tested for the search results (Table S3), and quality control was performed on the mass spectral data to determine whether the peptide lengths were mainly distributed within the range of 7-25 (Figure 2F). The mass deviation distribution of the measured molecular weight of the parent ion (primary mass spectrum, i.e., peptide ion) and theoretical molecular weight of the peptide was such that the peak types were concentrated near 0 (Figure 2G), indicating that the mass deviation of the peptide was small and that data quality of the peptide detected by mass spectrometry was good. Through protein database comparison, unique peptide fragments were identified (Figure 2H), and the curve increased slowly, indicating that the number of unique peptide fragments was large, that is, more reliable proteins were identified. The molecular weight distribution of the proteins and range of identified proteins was wide (Figure 2I), suggesting that the protein omics data were reliable and of good quality.

### Combined transcriptome and proteome analysis

**Figure 3:**
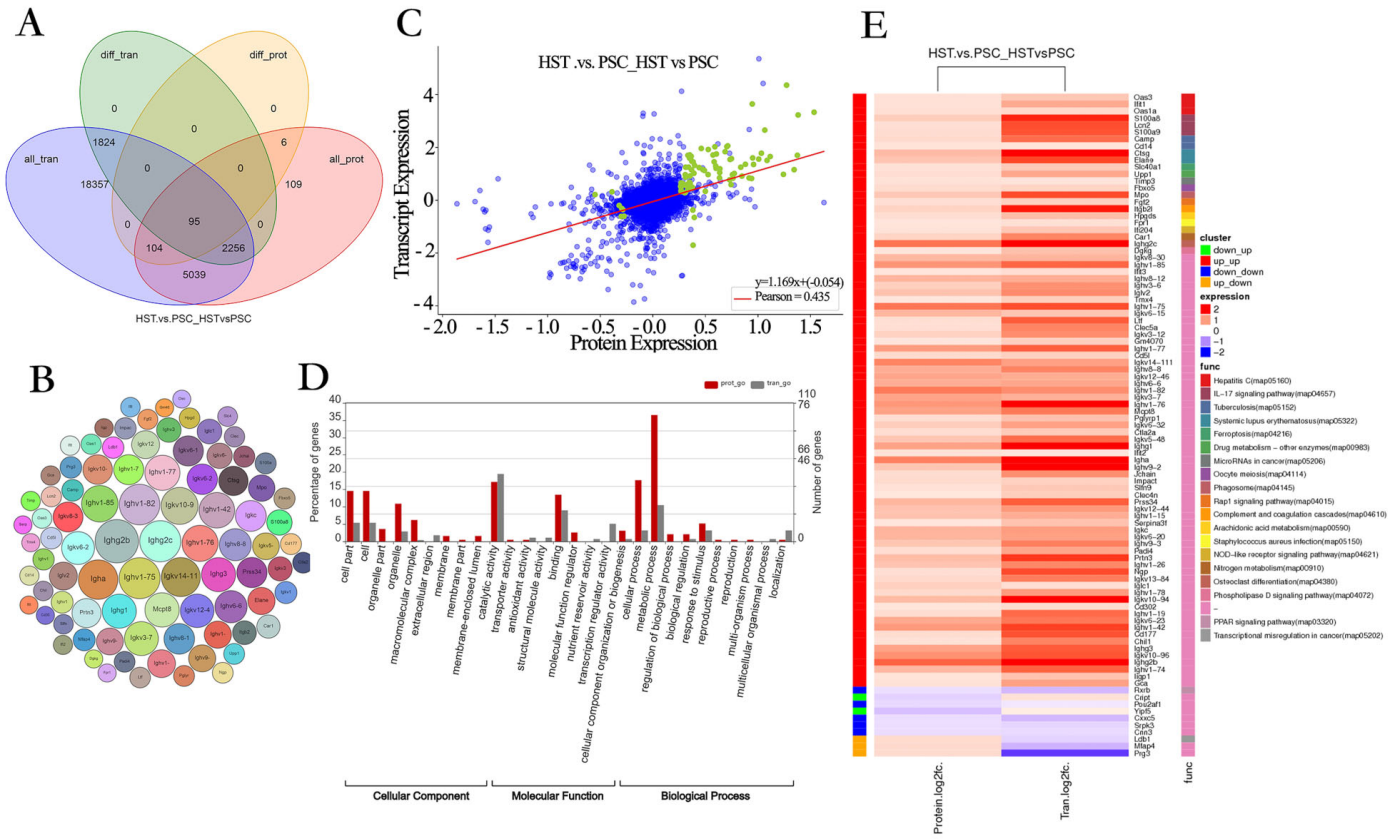
Gene expression analysis of mice in the high-altitude spleen test (HST) group and in the plain spleen control (PSC) group. **A**, Venn diagram of transcriptome and proteome expression regulation. all_tran represents all genes obtained from transcriptome, diff_tran represents differentially expressed genes identified from transcriptome, all_prot represents all proteins identified from proteome, diff_prot represents differentially expressed proteins identified from proteome. **B**, Bubble diagram of 95 prediction target gene. **C**, Association analysis of transcriptome and proteome expressions, each point in the graph represents a protein, green points represent proteins with significant differences in expression, blue points represent proteins with no significant differences in expression. **D**, Gene Ontology functional enrichment association analysis. **E**, Heat map of KEGG pathway enrichment clustering.

After 30 days of high-altitude hypoxia treatment, the mRNA information obtained from the transcriptome was integrated with the protein information identified in the protein group to determine the corresponding relationship. All-tran, diff-tran, all-prot, and diff-prot were selected, and the number of four database genes (proteins) was 27675, 4175, 7609, and 205, respectively. The Wayne diagram was drawn to take the intersection (Figure 3A), and a dataset of 95 predicted target genes (proteins) was obtained (Figure 3B), which was used as the aggregate set for subsequent analysis. To investigate the correlation between the data of the spleen transcriptome and the protein group treated with high-altitude hypoxia and normoxia, the multiple difference (log2 value) of the gene (protein) jointly identified by the transcriptome and the protein group in the two groups was analyzed for correlation (Figure 3C). The results suggested a significantly positive correlation between the transcription and protein omics expression levels in mouse spleen tissues at different altitudes.

To understand the biological function of DEGs (proteins) in the hypoxia stress response, GO enrichment analysis was performed on both transcriptome and protein groups (Figure 3D). The results showed that CC was mainly expressed in cells and macromolecular complexes, while MF was mainly expressed in functions such as catalytic activity and binding, and BP was mainly involved in metabolic, cellular, and stimulation reactions. Subsequently, the KEGG enrichment results for DEGs (proteins) in the transcriptional and protein groups were plotted on a KEGG pathway enrichment clustering heat map. Clustering was performed based on the differential expression multiple at the differential gene (protein) level, and 20 co-enriched KEGG joint pathways were successfully located (Figure 3E). The results indicated that DEGs (proteins) were highly enriched in multiple immune inflammation and lipid metabolism pathways, such as ferroptosis, phospholipase D, and NOD-like receptor signaling pathways, indicating that there may be important changes in the body under high-altitude hypoxic stress.

### GSEA based on KEGG functional annotation

**Figure 4:**
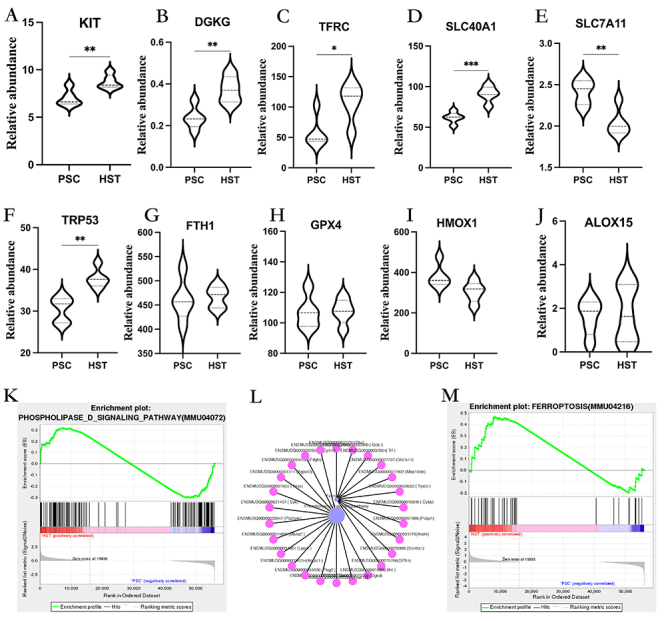
**A**-**J**, Relative expression of key genes in phospholipase D pathway and iron death pathway in mice in the high-altitude hypoxia (HST) group and in the plain normal oxygen (PSC) group (n=5/group). *P<0.05, **P<0.01, ***P<0.001 (Student's *t*-test). **K**, Enrichment plot: PHOSPHOLIPASE_D_SIGNALING_PATHWAY(MMU04072) Profile of the Running ES Score & Positions of GeneSet Members on the Rank Ordered List. **L**, Kegg advanced network diagram: purple circles represent pathways, red circles represent genes, black lines represent positive correlation. **M**, Enrichment plot: FERROPTOSIS(MMU04216) Profile of the Running ES Score & Positions of GeneSet Members on the Rank Ordered List.

Hypoxia can induce abnormal lipid metabolism, further induce ferroptosis, and mediate splenic injury. Therefore, we focused on lipid metabolism-related pathways, namely, the phospholipase D and ferroptosis pathways. Phospholipase D and ferroptosis were analyzed using GSEA. The relative expression trend of key genes in the two pathways was analyzed (Figure 4A-J), and further, in order to understand the interaction between the two pathways, the correlation between phospholipase D and ferroptosis signal pathway was analyzed. The results showed that there was a strong positive correlation between the two pathways (Figure 4L). GSEA results also showed that the enrichment score (ES) peak of phospholipase D and ferroptosis pathways appeared at the front end (ES value >0), indicating that gene expression of this pathway was mainly enriched in the HST group and the expression trend was generally upregulated (Figure 4K and M), which was consistent with the results of KEGG pathway enrichment analysis, indicating that phospholipase D and the ferroptosis pathway had important changes under hypoxic stress. To study the heterogeneity between the differential genes in the phospholipase D and ferroptosis pathways in the spleen transcriptome treated with high-altitude hypoxia and normoxia, the distribution of gene expression in the detected samples under the gene set was analyzed in the form of a heat map. The results showed that the gene expression profiles among the biological repeat samples were consistent and that the gene expression in the phospholipase D and ferroptosis pathways in mouse spleen tissues at different altitudes was different (Supplementary Figure S1A and B).

### Validation of differential genes and proteins in the phospholipase D signaling pathway

**Figure 5:**
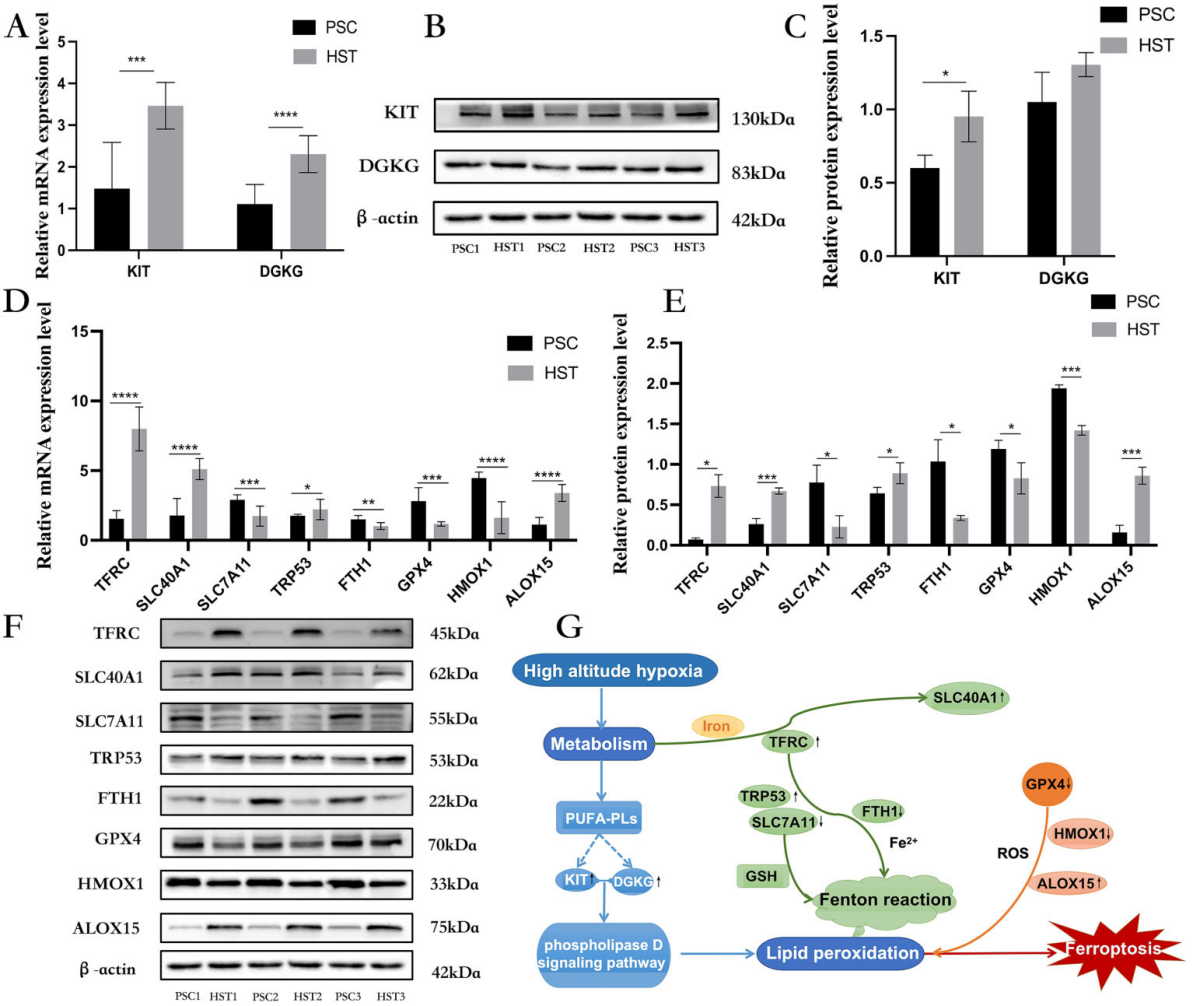
**A**-**C**, Quantitative analysis of mRNA expression, protein expression levels, and protein expression of KIT and DGKG in phospholipase D signaling pathway with β-actin as standard in mice in the high-altitude spleen test (HST) group and in the plain spleen control (PSC) group (n=3/group). **D**-**F**, Quantitative analysis of mRNA expression, protein expression levels, and protein expression of key genes in the ferroptosis signaling pathway, using β-actin as the standard (n=3/group). *P<0.05, **P<0.01, ***P<0.001, ****P<0.0001 (Student's *t*-test). **G**, Ferroptosis signaling pathway.

We further explored whether mouse spleen tissues exposed to hypoxia undergo changes related to lipid metabolism. Key phospholipase D genes were verified by real-time quantitative PCR (RT-qPCR) and western blotting. The mRNA and protein expressions of tyrosine kinase receptor *KIT* and diacylglycerol kinase *DGKG* in the phospholipase D signaling pathway were upregulated, and the protein expression of *DGKG* showed an upregulation trend without a significant difference (Figure 5A-C). The above results suggested that hypoxic stimulation induced the expression levels of phospholipase D signaling pathway-related genes and their encoded proteins in the spleen of mice, thus affecting lipid metabolism.

### Validation of differential genes and proteins in the ferroptosis signaling pathway

Based on the above results, we further analyzed the mRNA and protein expressions of DEGs in the ferroptosis signaling pathway. The results showed that the genes of the exogenous pathway such as *TFRC*, *SLC40A1*, *SLC7A11*, *TRP53*, and *FTH1*, and those of the endogenous pathway such as *GPX4*, *HMOX1*, and *ALOX15* showed significantly different changes between the PSC and HST groups (Figure 5D-F). Moreover, the protein expression levels were consistent with the gene expression levels. These results suggest that high-altitude hypoxia may mediate ferroptosis by affecting its signaling pathway (Figure 5G).

## Discussion

This study developed an animal model of high-altitude hypoxia and, based on a combined analysis of transcriptome and protein data, explored the mechanisms of lipid metabolism and ferroptosis in mouse spleens under high-altitude hypoxia. Our results showed that the mRNA and protein expression of *HIF-1α* in mouse spleen tissues was significantly increased in the HST group compared with the PSC group, suggesting that the hypoxia animal model was successfully developed. Changes in spleen weight reflect the body's immune function to some extent. Our results also indicated that the body weight, spleen weight, and spleen index of mice in the high-altitude hypoxia group were significantly decreased, suggesting that spleen tissues were altered under high-altitude hypoxia stress.

Functional enrichment analysis of the predicted target genes in this study showed that the DEGs were enriched in ferroptosis, phospholipase D, PPAR, IL-17, and NOD-like receptor signaling pathways. Ferroptosis is an iron-dependent non-apoptotic form of cell death (13). Hypoxia activates NADPH cytochrome P450 reductase and NADH cytochrome b5 reductase on the endoplasmic reticulum membrane to generate H_2_O_2_ and react with ferrous ions in a Fenton reaction, thus triggering the lipid free radical chain reaction and generating a large number of lipid free radicals that attack more membrane phospholipids to form a lipid oxidation cascade (14-16). Subsequently, the molecular structure of the phospholipid membrane is damaged (17), and the cumulative damage to the membrane structure leads to the formation of cell membrane pores, which is a landmark event in ferroptosis (18). Therefore, we focused on changes in phospholipase D and ferroptosis pathway-related genes under hypoxic stimulation. The results of gene set enrichment analysis showed that the upregulated DEGs were significantly enriched in phospholipase D and ferroptosis signaling pathways, which was consistent with the enrichment results of the KEGG pathway. These results suggested that hypoxic stimulation promoted ferroptosis by inducing changes in the phospholipase D pathway in the mouse spleen.

Membrane damage induced by phospholipid oxidative stress is key to the induction of ferroptosis (19). Therefore, in this study, the DEGs *KIT* and *DGKG* in the phospholipase D signaling pathway were used to explore the mechanism of their participation in splenic phospholipid metabolism under hypoxic stimulation. After binding to its ligand *SCF*, *KIT* undergoes homodimer transformation, which activates the PI3K/AKT axis to participate in the growth, proliferation, and differentiation of gastrointestinal stromal cells (20). *KIT* overexpression abnormally activates mast cell degranulation, resulting in the release of pro-inflammatory cytokines and immune inflammatory response (21). As a precursor of phosphatidic acid, *DGKG* is a key kinase that maintains the metabolic balance of phospholipids (22,23). In the early stage of hypoxia-ischemia, *DGKG* is rapidly transferred from the nucleus to the cytoplasm, causing impaired neurological function (neuronal degeneration or apoptosis) (24). The toxic accumulation of phospholipid peroxide formed by the peroxidation of phospholipid PUFA on the cell membrane drives ferroptosis (25). Accordingly, our study further validated the mRNA and protein expression levels of the *KIT* and *DGKG* genes in the phospholipase D signaling pathway. The results indicated an upregulation of *KIT* and *DGKG* in spleen tissues in the HST group compared with the PSC group, suggesting that high-altitude hypoxia affected ferroptosis in the body by activating the phospholipase D metabolic pathway.

To further explore the regulatory mechanism of the ferroptosis signaling pathway in spleen tissues under hypoxic stress, we screened for significantly different genes with high expression. The transferrin receptor (*TFRC*) and iron transporter (*SLC40A1*) are essential for maintaining intracellular iron concentrations. Fe^3+^ enters cells through *TFRC* and is reduced to Fe^2+^ in the lysosomes. After entering the free iron pool, Fe^2+^ promotes the formation of lipid peroxide and leads to free iron overload (26). *SLC40A1* is a multi-transmembrane protein and the only known mammalian iron transporter that transports iron from the cytoplasm to the extracellular space (27). This study showed that the expression of *TFRC* and *SLC40A1* was upregulated under hypoxic stimulation, which was speculated to be due to the increase in cellular iron, leading to the efflux of iron ions under oxidative stress, thereby relieving the intracellular iron overload and inducing *SLC40A1* overexpression. *SLC7A11* is the main transporter of intracellular glutamic acid and extracellular cystine (i.e., the cystine/glutamic acid reverse transporter) and plays a key role in maintaining cell survival under oxidative stress conditions (28,29). Activated *TRP53* protein can bind to the promoter region of the *SLC7A11* gene, inhibit *SLC7A11* expression, reduce cystine uptake, and limit glutathione production, resulting in cell damage (30,31). In this study, the expression of *TRP53* increased, whereas that of *SLC7A11* decreased, suggesting that hypoxia caused a decrease in cell antioxidant capacity through *TRP53*-mediated downregulation of *SLC7A11*, thus promoting ferroptosis. Additionally, we found that the expression of ferritin heavy chain 1 (*FTH1*) decreased under hypoxic conditions. *FTH1* is mainly involved in the regulation of iron-ion storage. The reduced expression of *FTH1* increases free iron levels, causing the Fenton reaction and promoting ferroptosis (32). Dihydroartemisinin accelerates the degradation of ferritin by inducing autophagy, resulting in an increase in ROS content in cells (33). Knockout of *FTH1* significantly inhibits cell viability, leading to mitochondrial dysfunction (34).

This study also showed that hypoxia inhibited the mRNA and protein expression levels of *GPX4*, suggesting that *GPX4* may cause ferroptosis induced by the accumulation of lipid peroxides in the spleen. Heme oxygenase-1 (*HMOX1*), an antioxidant enzyme, plays an important role in protecting cells from oxidative stress injury induced by external stimuli (35). Additionally, *Nrf2*/*Hmox1* axis-mediated mitochondrial iron accumulation and phospholipid peroxidation can lead to ferroptosis in myocardial cells in rat heart ischemia-reperfusion injury (36). In this study, *HMOX1* expression decreased, suggesting that *HMOX1* played a role in hypoxia-induced ferroptosis in the spleen. Additionally, we found an increased expression of arachidonic acid-15-lipoxygenase (*ALOX15*) under hypoxic exposure. *ALOX15*, a member of the arachidonic acid lipoxygenase family (37), is considered a key mediator of phospholipid peroxidation attack and mediation of mitochondrial dysfunction (38). Phosphatidyl ethanolamine binding protein-1 (PEBP1), a chaperone of *ALOX15*, can rivet *ALOX15* to the plasma membrane, promoting peroxidation damage of phosphatidyl ethanolamine (PE) in the plasma membrane and triggering ferroptosis (39).

High-altitude hypoxia stimulation mediates phospholipid metabolism in spleen tissues and often causes ferroptosis. Therefore, identifying the key genes responsible for the induction of phospholipid metabolism and ferroptosis under high-altitude hypoxia provides clues for the molecular regulation of lipid metabolism disorders and ferroptosis induced by high-altitude hypoxia.

### Conclusion

Our results suggested that hypoxia induced ferroptosis in splenic tissues by regulating the exogenous transporter protein-dependent, endogenous enzyme-regulated, and phospholipase D pathways, leading to the accumulation of phospholipid peroxides. This study provided insights into how hypoxic stimulation at the plateau regulates phospholipid metabolism and ferroptosis processes in spleen tissues and, thus, provided new ideas and directions for the mechanism of hypoxia-induced splenic injury.

## Supplementary Material

Click here to view [pdf].
