# Supplementary material for: Phospholipase D regulates ferroptosis signal transduction in mouse spleen hypoxia response
Source: Braz J Med Biol Res. 2024 Mar 4;57:e13218. doi: 10.1590/1414-431X2023e13218 (PMC10913393; doi:10.1590/1414-431X2023e13218)
Supplement: Supplementary file 1 [file 1414-431X-bjmbr-57-e13218-suppl.pdf]

Figure S1. Phospholipase D and ferroptosis GSEA enrichment analysis graph. **A**, PHOSPHOLIPASE_D_SIGNALING_PATHWAY(MMU04072) Blue-Pink O' Gram in the Space of the Analyzed GeneSet. **B**, FERROPTOSIS (MMU04216) Blue-Pink O' Gram in the Space of the Analyzed GeneSet.

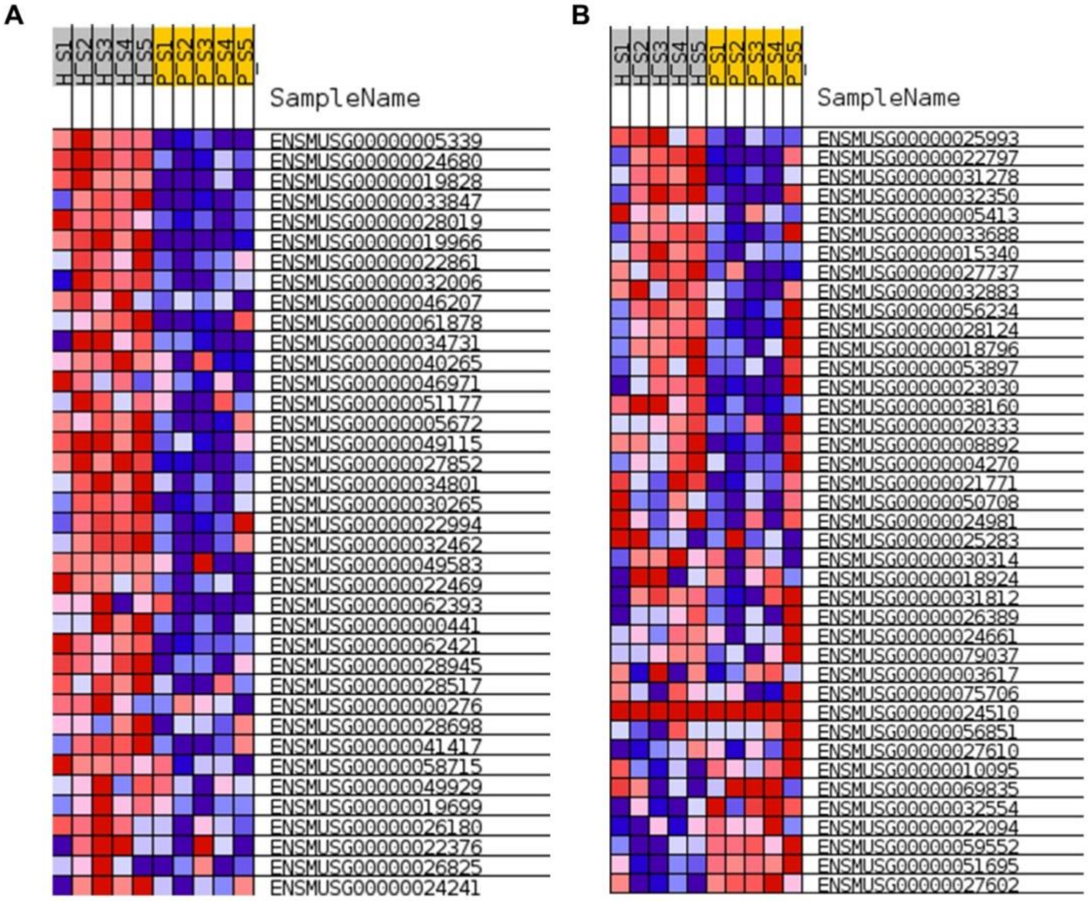

Table S1. Primer information of the genes for RT-qPCR.

Gene	Primer sequence	bp
β-Actin	F: CATCCGTAAAGACCTCTATGCCAAC	25
	R: ATGGAGCCACCGATCCACA	19
KIT	F: GATCTGCTCTGCGTCCTGTTGG	22
	R: AACTCTGATTGTGCTGGATGGATGG	25
DGKG	F: TGACGGAGTTGAGGTGGATCTGAG	24
	R: CTGTGACGCTCTTCCTGCTTTCC	23
TFRC	F: TCGTGGAGACTACTTCCGTGCTAC	26
	R: TCTTGGAGATACATAGGGCGACAGG	27
SLC40A1	F: TTGGTGACTGGGTGGATAAGAATGC	27
	R: CGCAGAGGATGACGGACACATTC	25
SLC7A11	F: TGTGTTCGCTGTCTCCAGGTTATTC	27
	R: GAGAAGAGCATCACCATCGTCAGAG	27
TRP53	F: ACCGCCGACCTATCCTTACCATC	25
	R: GGCACAAACACGAACCTCAAAGC	25
FTH1	F: TGCCATCAACCGCCAGATCAAC	24
	R: AGTTCTTCAGAGCCACATCATCTCG	27
GPX4	F: ATAAGAACGGCTGCGTGGTGAAG	25
	R: TAGAGATAGCACGGCAGGTCCTTC	26
HMOX1	F: ACCGCCTTCCTGCTCAACATTG	24
	R: CTCTGACGAAGTGACGCCATCTG	25
ALOX15	F: GGAAGAAAGGAGGAGTCTGTAC	22
	R: GTCTTTTGTCCTCTCGAAATCG	22

Table S2. Quality statistics of sample sequencing data.

Sample	Raw reads	Clean reads	Clean bases	Error rate	Q20 (%)	Q30 (%)	GC (%)
P_S1	45514564	43655862	6.55G	0.02	98.31	95.07	50.34
P_S2	44831974	42944652	6.44G	0.02	98.21	94.81	49.69
P_S3	45847080	44054100	6.61G	0.02	98.13	94.68	50.26
P_S4	41626856	40090630	6.01G	0.02	98.37	95.17	50.18
P_S5	42402928	41319526	6.20G	0.02	98.33	95.01	50.33
H_S1	41251434	40011482	6.00G	0.02	98.23	94.85	49.48
H_S2	41666006	40187820	6.03G	0.02	98.28	94.93	48.45
H_S3	44130378	42116220	6.32G	0.02	98.42	95.25	48.28
H_S4	44856906	43106466	6.47G	0.02	98.25	94.86	48.89
H_S5	44549044	42632818	6.39G	0.02	98.32	95.01	48.65

P: plain spleen control group; H: high-altitude spleen test group.

Table S3. Proteome Discoverer search database analysis parameters.

Item	Value
Type of quantification	Reporter quantification (TMT)
Enzyme	Trypsin
Maximum missed cleavage sites	2
Precursor mass tolerance	10 ppm
Fragment mass tolerance	0.02 Da
Dynamic modification	Oxidation/+15.995 Da (M) and TMT pro/+304.207 Da (K)
N-terminal modification	Acetyl/+42.011 Da (N-terminal) and TMT pro/+304.207 Da (N-terminal) and Met-loss/–131.040Da(M) and Met-loss+acetyl/–89.030 Da(M)
Static modification	Carbamidomethyl/+57.021 Da (C)
